# Prime-Pull Immunization with a Bivalent M-Protein and Spy-CEP Peptide Vaccine Adjuvanted with CAF®01 Liposomes Induces Both Mucosal and Peripheral Protection from *covR/S* Mutant Streptococcus pyogenes

**DOI:** 10.1128/mBio.03537-20

**Published:** 2021-02-23

**Authors:** Victoria Ozberk, Simone Reynolds, Yongbao Huo, Ainslie Calcutt, Sharareh Eskandari, Jessica Dooley, Jamie-Lee Mills, Ida S. Rasmussen, Jes Dietrich, Manisha Pandey, Michael F. Good

**Affiliations:** a Institute for Glycomics, Griffith University, Gold Coast, Australia; b Center for Vaccine Research, Statens Serum Institut, Copenhagen, Denmark; Emory University School of Medicine

**Keywords:** *Streptococcus pyogenes*, mucosal adjuvants, mucosal vaccines, toxicology, vaccines

## Abstract

Infections with Streptococcus pyogenes and their sequelae are responsible for an estimated 18 million cases of serious disease with >700 million new primary cases and 500,000 deaths per year. Despite the burden of disease, there is currently no vaccine available for this organism. Here, we define a combination vaccine P*17/K4S2 comprising of 20-mer B-cell peptide epitopes, p*17 (a mutant derived from the highly conserved C3-repeat region of the M-protein), and K4S2 (derived from the streptococcal anti-neutrophil factor, Spy-CEP). The peptides are chemically conjugated to either diphtheria toxoid (DT) or a nontoxic mutant form of diphtheria toxin, CRM197. We demonstrate that a prime-pull immunization regimen involving two intramuscular inoculations with P*17/K4S2 adjuvanted with a two-component liposomal adjuvant system (CAF01; developed by Statens Serum Institut [SSI], Denmark), followed by an intranasal inoculation of unadjuvanted vaccine (in Tris) induces peptide- and S. pyogenes-binding antibodies and protects from mucosal and skin infection with hypervirulent *covR/S* mutant organisms. Prior vaccination with DT does not diminish the response to the conjugate peptide vaccines. Detailed Good Laboratory Practice (GLP) toxicological evaluation in male and female rats did not reveal any gross or histopathological adverse effects.

## INTRODUCTION

Streptococcus pyogenes (group A streptococcus) is a human pathogen that primarily infects the skin and oropharynx, resulting in mild and mostly self-resolving conditions. However, bacteria often disseminate to normally sterile sites within the body and this can lead to invasive disease that is associated with high morbidity and mortality. Repeated episodes of S. pyogenes infection can cause the post-streptococcal sequelae of rheumatic fever (RF), rheumatic heart disease (RHD), and acute post-streptococcal glomerulonephritis (ASPGN) (1). Globally there are more than 30 million cases of RHD causing more than 300,000 deaths each year (2). The WHO and World Heart Federation have called for a 25% reduction in mortality due to cardiovascular causes, including RHD, by 2025 (3).

Immunity to S. pyogenes in humans takes years to develop. Its pathogenesis derives from virulence factors that subvert innate and acquired immunity (4) and by the fact that its dominant antigen, the M-protein, is highly polymorphic at its amino terminus (∼250 serotypes) (5, 6). This has severely hindered vaccine development. We described a 20-mer B-cell peptide epitope, p*17, based on the highly conserved C3-repeat region of the M-protein. It has two non-natural mutations relative to the native sequence (7). These result in the peptide maintaining a stable alpha helical conformation and is associated with significantly enhanced immunogenicity (7). However, organisms that have mutations within *covR/S* are highly virulent due to the upregulation of various virulence factors, including the neutrophil anti-chemotaxis factor, Spy-CEP. Antibodies that target the C3-repeat region of the M-protein require neutrophils for anti-streptococcal activity (8). Thus, in order to improve the efficacy of a C3-repeat region-based vaccine, we identified a highly conserved 20-mer epitope, S2 (or K4S2 [S2 with four lysine residues added to improve solubility]), from Spy-CEP and combined it with p*17. Mice vaccinated with the combination vaccine (p*17 with K4S2, each component individually conjugated to diphtheria toxoid [DT]) formulated in aluminum hydroxide [Al[OH]_3_] [Alum]) demonstrated a significant reduction in bacterial burden in skin and blood following skin challenge with *covR/S* mutant organisms (9).

While an Alum-formulated vaccine delivered intramuscularly (i.m.) induced site-specific immunity that protected against skin and invasive infection, it showed no efficacy against upper respiratory tract (URT) S. pyogenes infection (10). In an effort to address this issue, we utilized a recently developed human-approved liposome-based delivery system as a vaccine adjuvant. CAF®01 is a two-component liposomal adjuvant system composed of cationic liposome N′,N-dimethyl-N,N′-dioctadecylammonium (DDA) bromide stabilized with the synthetic mycobacterial immunomodulator α,α′-trehalose 6,6′-dibehenate (TDB), which is a synthetic variant of the cord factor located in the mycobacterial cell wall. In addition to a strong systemic response, a “prime-pull” (intramuscular [i.m.] immunization on days 0 and 21 and an intranasal [i.n.] immunization on day 42) vaccination strategy with CAF®01 promoted the stimulation of a local mucosal Th17 response and protection against infection with S. pyogenes (11, 12). CAF®01 was assessed for safety and immunogenicity in clinical trials involving a tuberculosis (TB) vaccine (13), an HIV-1 peptide cocktail vaccine (14), a recombinant malaria vaccine (15), and a chlamydia vaccine (16). Induction of mucosal and systemic immunity was shown following a prime-pull immunization regimen and CAF®01 was reported as both safe and well tolerated (13–16).

The combination vaccine involving two B-cell epitopes from the two major virulence factors, M-protein and Spy-CEP, formulated with the mucosal adjuvant CAF®01, is called P*17/K4S2. The peptides are individually conjugated to either DT, as P*17/K4S2(DT), or to the closely related mutant diphtheria toxin, CRM197, as P*17/K4S2(CRM). Here, we assessed the immunogenicity and efficacy of P*17/K4S2 in mice and present the results of a formal GLP toxicological evaluation of P*17/K4S2(CRM) in rats.

## RESULTS

### Prime-pull immunization with P*17/K4S2(DT) and P*17/K4S2(CRM) induces mucosal and systemic antibody responses and protects against URT S. pyogenes infection.

**FIG 1:**
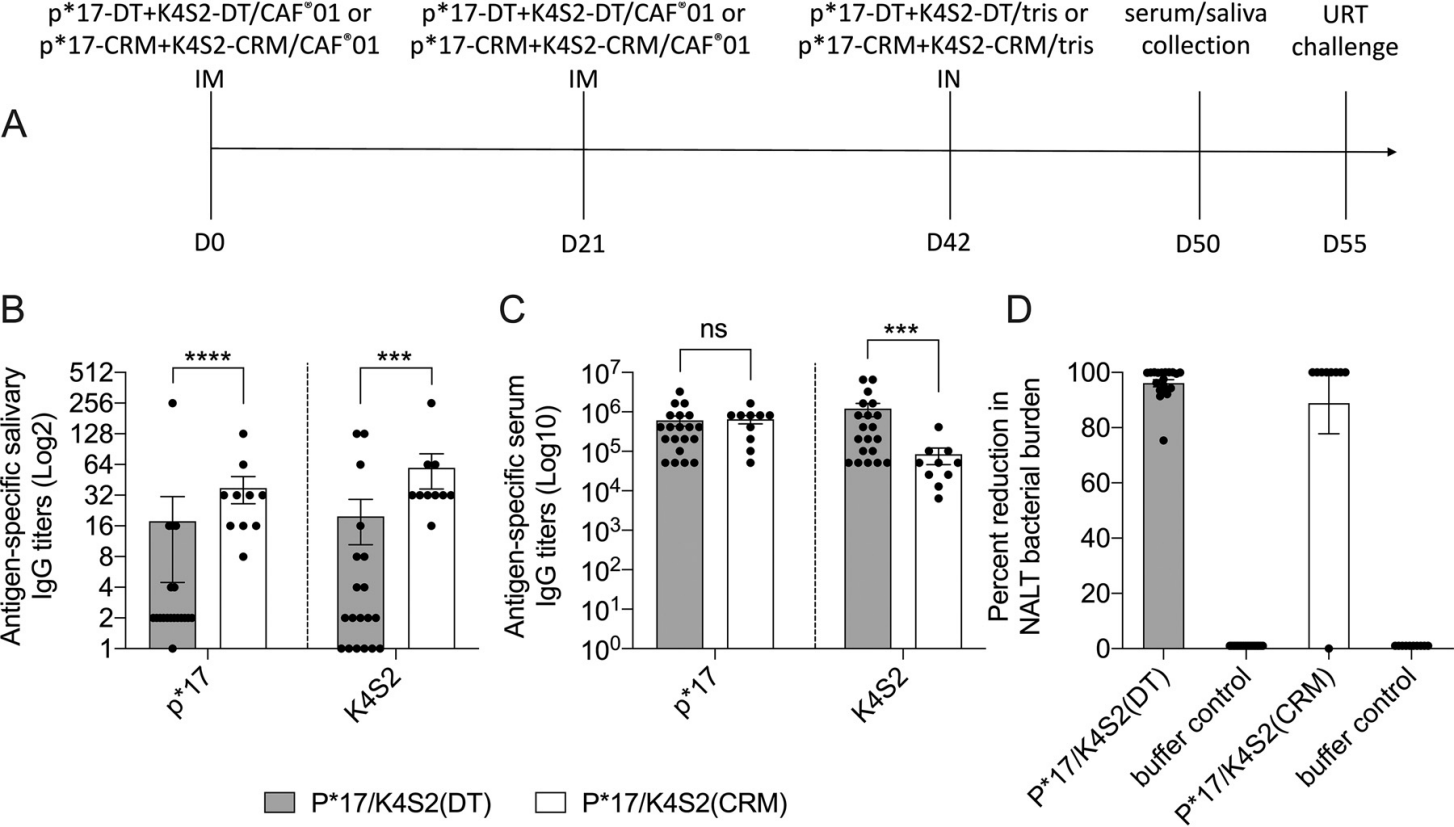
Immunogenicity and protective efficacy following prime-pull immunization with P*17/K4S2(DT) and P*17/K4S2(CRM). BALB/c mice were immunized via the prime-pull method with P*17/K4S2(DT) (*n* = 20) or P*17/K4S2(CRM) (*n* = 9) (day 0 and 21, i.m. with antigen/CAF®01; day 42, i.n. with antigen/Tris) and Tris only (day 0 and 21, i.m. with Tris; day 42, i.n. with Tris). (A) Immunization, sample collection, and challenge timeline. (B and C) Immunogenicity of vaccines. Serum and saliva samples were collected one week post last vaccine boost and antigen-specific antibody levels were measured by ELISA. The endpoint titer was defined as the highest dilution that gave an absorbance of >3 standard deviations above the mean absorbance of negative-control wells. Salivary IgG titers (B) and serum IgG titers (C) for individual mice are shown. Data are mean ± standard error of the mean (SEM). Statistical comparisons were performed using the Mann-Whitney test in GraphPad Prism 8.1.2: ns, *P* > 0.05; ***, *P* < 0.001; ****, *P* < 0.0001. (D) Percent reduction in NALT bacterial burden. Mice were challenged intranasally with the *covR/S* MT S. pyogenes isolate, 5448AP. On day 3 post-infection, all mice were sacrificed and organs harvested for assessment of bacterial load. Percent reduction in NALT bacterial load for each individual mouse in the vaccinated cohort was calculated in comparison to the mean bacterial burden of buffer control cohort. The data are from two independent experiments with either P*17/K4S2(DT) or P*17/K4S2(CRM). The mean bacterial burden (CFU/ml) for the corresponding buffer controls (DT and CRM experiments) were 4.12 × 10^12^ and 3.95 × 10^4^, respectively. We chose to use a Tris (buffer) control for these experiments; however, preliminary experiments using CAF®01/Tris as a control (2× i.m. injections with CAF®01 and 1× i.n. with Tris) showed that vaccination with P*17/K4S2(CRM) (2× i.m. injections with p*17-CRM+K4S2-CRM/CAF®01 and 1× i.n. with p*17-CRM+K4S2-CRM/Tris) resulted in >88% protection in NALT (judged by colony count reduction).

The vaccine delivery system and route of administration are critical for the development of immune responses that may correlate with site-specific or cross-compartment protection (17, 18). To induce both mucosal and systemic immunity, we utilized a prime-pull vaccination strategy (Fig. 1A). Naïve BALB/c mice were immunized via the i.m. route on days 0 and 21 with vaccine in CAF®01 and on day 42 i.n. with vaccine in Tris buffer (no CAF®01). We compared P*17/K4S2(DT) and P*17/K4S2(CRM). Serum and saliva samples were collected on day 50 and antigen (p*17 and K4S2)-specific salivary IgG (Fig. 1B) and serum IgG (Fig. 1C) were assessed by enzyme-linked immunosorbent assay (ELISA). There was insufficient saliva to test IgA. Mice vaccinated with P*17/K4S2(CRM) had significantly higher p*17- and K4S2-specific salivary IgG titers than mice vaccinated with P*17/K4S2(DT) (Fig. 1B). Both vaccines induced comparable serum IgG responses for p*17; however, P*17/K4S2(DT) induced significantly higher K4S2-specific IgG in comparison to P*17/K4S2(CRM) (Fig. 1C). P*17/K4S2(DT) was also shown to be highly immunogenic in HLA DR3-DQ2-humanized mice (Fig. S1A in the supplemental material). At 2 weeks post final vaccination (day 56), mice were challenged i.n. with the *covR/S* mutant (MT) S. pyogenes isolate 5448AP and the protective efficacy of the vaccine was determined. Both vaccines protected mice, as shown by a 96% and 87% reduction in bacterial colony counts in the nasal-associated lymphoid tissues (NALT) in comparison to the corresponding buffer control cohorts (Fig. 1D).

### Preexisting carrier-specific antibodies do not affect vaccine peptide-specific antibody responses.

**FIG 2:**
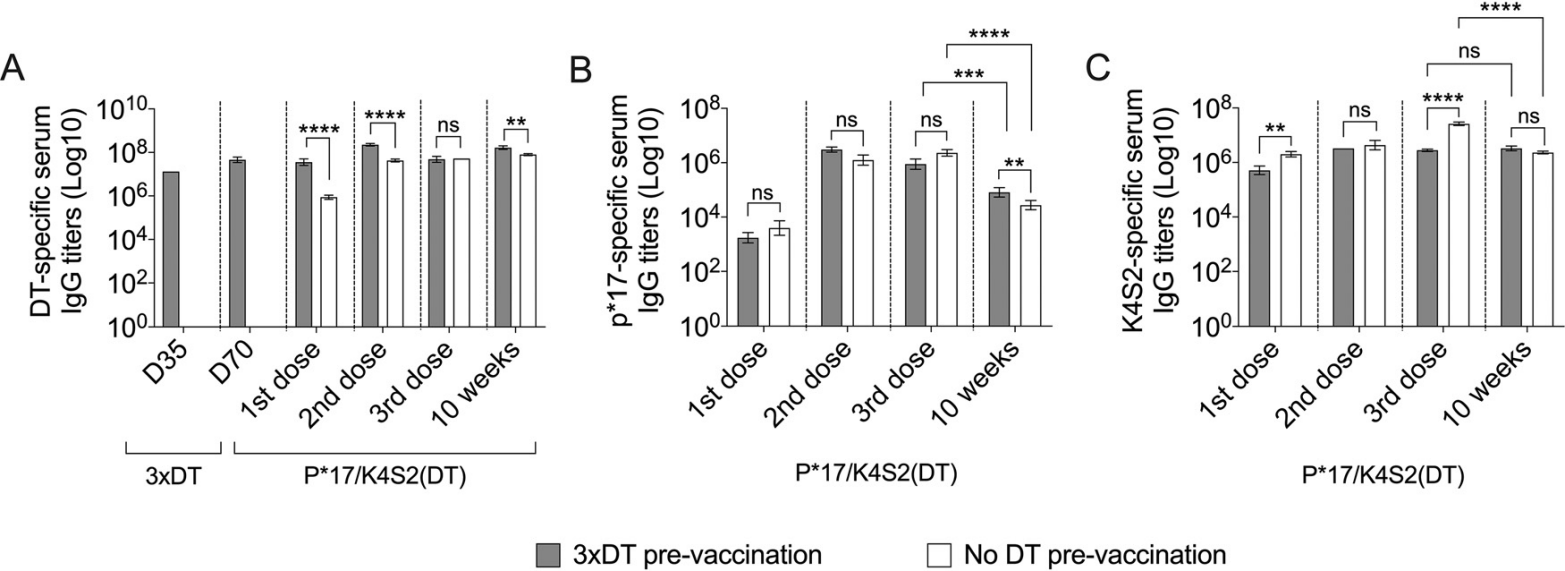
DT preexposure and vaccine immunogenicity. BALB/c mice (*n* = 10) were immunized i.m. with DT/Alum (3× DT prevaccination) on days 0, 21, and 28 or left unvaccinated (no DT prevaccination). Serum was collected on days 35 (1 week post last DT exposure) and 70 (5 weeks post last DT exposure) to assess DT-specific antibodies. Following DT exposure, mice were vaccinated with P*17/K4S2(DT) using the prime-pull immunization regimen. Serum samples were collected after each vaccination (up to 10 weeks) and antigen-specific antibody titers were followed by ELISA. (A) DT-specific serum IgG titers. (B) p*17-specific serum IgG titers. (C) K4S2-specific serum IgG titers. The endpoint titer was defined as the highest dilution that gave an absorbance of >3 standard deviations above the mean absorbance of negative control wells. Data are mean ± SEM of *n* = 10 mice/group. Statistical analysis was performed using a nonparametric, unpaired Mann-Whitney U test (one-tailed) to compare 3×DT prevaccination to the no DT prevaccination control group (ns, *P* > 0.05; **, *P* < 0.01; ***, *P* < 0.001; ****, *P* < 0.0001). Statistical analysis and graphs were generated in GraphPad Prism 8.1.2.

Preexisting immunity against a vaccine carrier can inhibit the immune system from recognizing vaccine antigen conjugated to the same carrier (19). This phenomenon is referred to as carrier-induced epitopic suppression (CIES) (19). We investigated whether preexisting DT-specific antibodies could suppress the immunogenicity of vaccine peptides in P*17/K4S2(DT). BALB/c mice were immunized with DT/Alum (3×DT prevaccination) or left unvaccinated (no DT prevaccination). At 5 weeks post DT exposure, mice were vaccinated via the prime-pull regimen with P*17/K4S2(DT). After the first vaccine dose, DT-preexposed mice had significantly higher DT-specific IgG levels in comparison to DT-unprimed (naive) mice (Fig. 2A). This significant difference was also seen following the second vaccine dose and also at 10 weeks postvaccination (Fig. 2A). No significant difference between p*17-sepcific (Fig. 2B) and K4S2-specific (Fig. 2C) IgG titers in DT-preexposed and DT-naive mice was noted. At 10 weeks post-vaccination, p*17-specific IgG levels in both DT-preexposed and DT-naive cohorts dropped compared to IgG levels after the third vaccine dose (Fig. 2B). However, for K4S2-specific IgG titers at the same time point, a significant drop was only seen in the DT-naive cohort (Fig. 2C). These data confirm that preexisting DT-specific antibodies do not affect p*17 and K4S2 immunogenicity.

### Vaccine-induced serum IgG recognizes and binds to S. pyogenes isolates.

**FIG 3:**
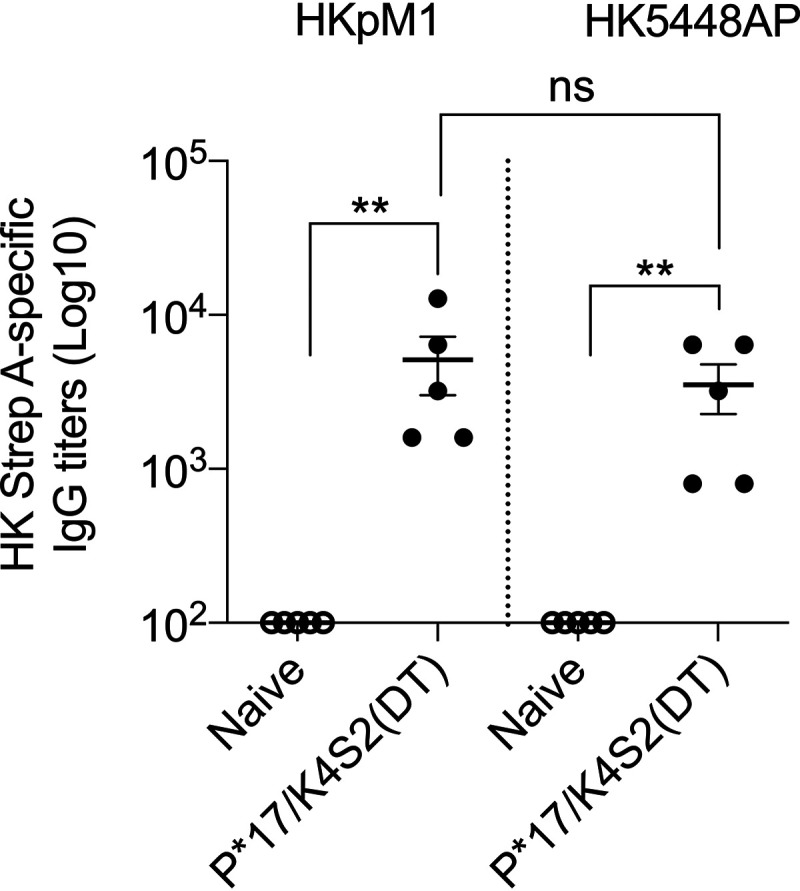
Binding of P*17/K4S2(DT) antisera to S. pyogenes. The binding of anti-P*17/K4S2(DT) and naive sera were assessed against heat-killed pM1 (HKpM1; *covR/S* WT) and heat-killed 5448AP (HK5448AP; *covR/S* MT) using whole-cell ELISA. Vaccine antibodies specific for HKpM1 and HK5448AP were detected using HRP-conjugated mouse secondary IgG antibody. The endpoint titer was defined as the highest dilution that gave an absorbance of >3 standard deviations above the mean absorbance of negative control wells. Data for 5 mice (± SEM) in vaccinated and control cohort are shown. Statistical analysis was performed using a nonparametric, unpaired Mann-Whitney U test (one-tailed) to compare naive antisera with P*17/K4S2(DT) antisera (**, *P* < 0.01). Statistical analysis and graphs were generated in GraphPad Prism 8.1.2.

We used a whole-cell ELISA to assess the binding of P*17/K4S2(DT)-induced antibodies to the surface of heat-killed pM1 (HKpM1; *covR/S* wild type [WT]) and heat-killed 5448AP (HK5448AP; *covR/S* mutant [MT]). P*17/K4S2(DT) antisera showed significantly higher IgG binding to HKpM1 and HK5448AP in comparison to naive sera (Fig. 3). The binding of antisera to HKpM1 and HK5448AP were comparable (Fig. 3).

### Vaccination with P*17/K4S2(DT) induces long-term protective immunity against URT and skin challenge infections.

**FIG 4:**
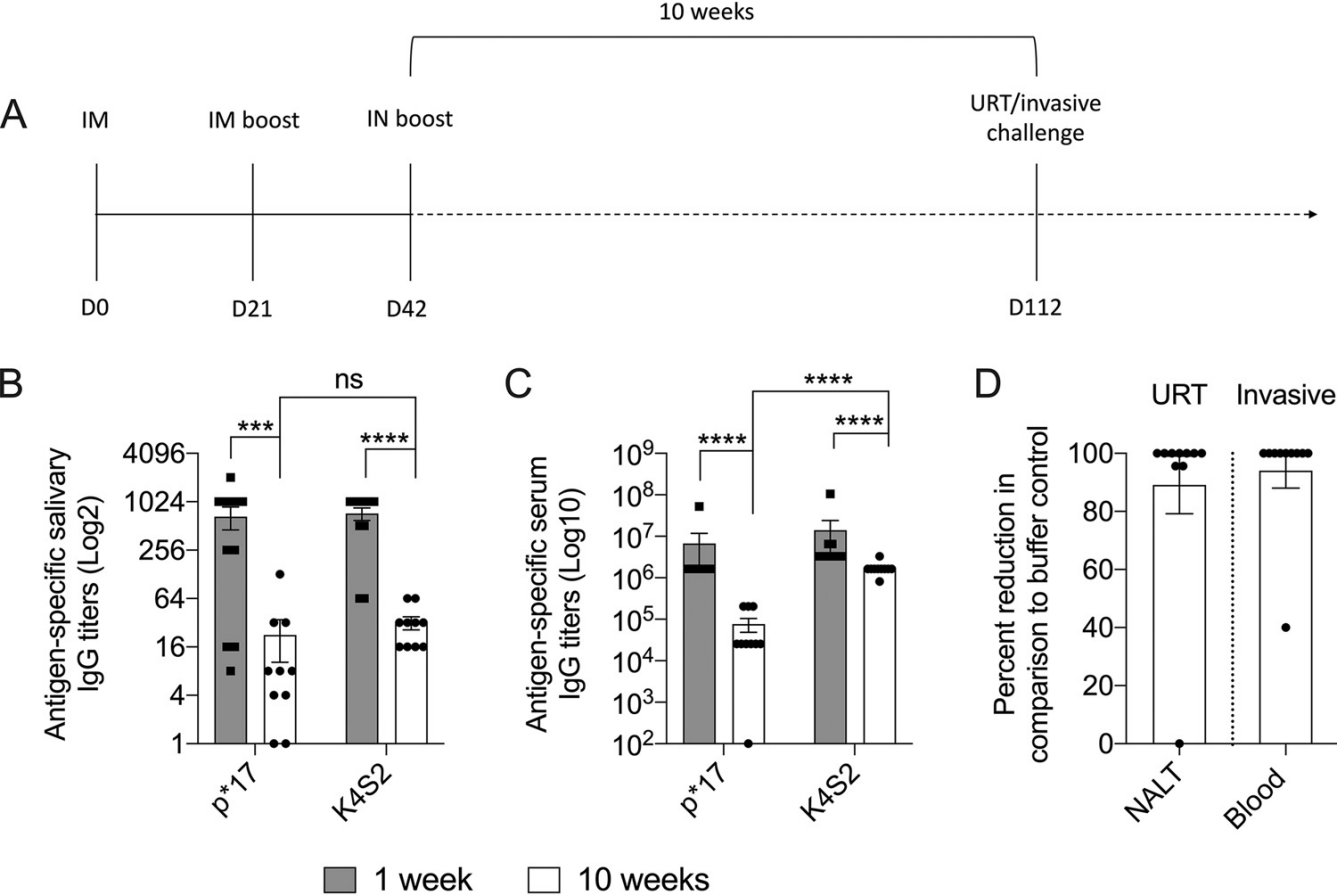
Longevity of immune responses 10 weeks following prime-pull immunization with P*17/K4S2(DT). BALB/c mice were immunized using the prime-pull immunization regimen with P*17/K4S2(DT) or 10 mM Tris buffer (buffer control). (A) Immunization and challenge timeline. (B and C) Longevity of vaccine-induced Ig response. Serum and saliva samples were collected 10 weeks after the last vaccine boost and antigen-specific IgG levels in saliva (B) and serum (C) were measured by ELISA. The endpoint titer was defined as the highest dilution that gave an absorbance of >3 standard deviations above the mean absorbance of negative control wells. Data for *n* = 10 mice as mean ± SEM are shown. Statistical comparisons were performed using a Mann-Whitney test (ns, *P* > 0.05; ***, *P* < 0.001; ****, *P* < 0.0001). (D) Assessment of longevity of vaccine-induced protective immunity. Mice were challenged intranasally or via the skin with *covR/S* MT S. pyogenes isolate 5448AP. Mice were sacrificed and percent reduction for each individual mouse in the vaccinated cohort was calculated in comparison to the mean of the buffer control cohort. Percent reduction in NALT bacterial burden (day 3) and percent reduction in blood bacterial burden (day 6) following URT and skin challenge infections, respectively, are shown (D). The mean bacterial burden (CFU/ml) for corresponding buffer controls (for the URT and invasive infections) were 5.7 × 10^2^ and 4 × 10^2^, respectively.

To assess if vaccination with P*17/K4S2(DT) led to long-term antibody memory and multicompartment protection, additional immunization and protection studies were undertaken. Mice were immunized with P*17/K4S2(DT) or 10 mM Tris buffer (buffer control) using the prime-pull method and rested for 10 weeks (Fig. 4A). In serum (Fig. 4B) and saliva (Fig. 4C), there was a significant drop in p*17- and K4S2-specific IgG titers at 1 versus 10 weeks post-immunization. At 10 weeks after the last vaccine boost, mice were challenged with the *covR/*S MT S. pyogenes isolate 5448AP either via the URT or skin route. On day 3 post-URT challenge, vaccinated and control mice were culled and NALT was removed for bacterial burden enumeration. Vaccinated mice showed >85% reduction in bacterial burden in NALT compared to the buffer control cohort (Fig. 4D). On day 6 post skin challenge, blood was collected to assess the effect of vaccination on invasive infection. We observed that vaccinated mice had a 94% reduction in bacteremia compared to the buffer control cohort (Fig. 4D). We could thus demonstrate effective immunity at both mucosal and skin portals of infection.

### GLP toxicology in Sprague-Dawley rats.

We used rats to determine the safety and immunogenicity of P*17/K4S2(CRM) following a prime-pull immunization regimen (2 × i.m. and 1 × i.n. dose) in Sprague-Dawley rats over a 6-week dosing period and a 4-week recovery period. The vaccine, control antigen (CRM), and vehicle (CAF®01 or Tris) were administered to male and female Sprague-Dawley rats (*n* = 20, 10/sex/group) on days 0, 21, and 42, as per Table 1 and Table 2. On days 0 and 21, formulations were administered as a 0.25 ml i.m. injection (alternating thighs). On day 42, formulations were administered (0.1 ml) by the i.n. route (Table 1 and 2). Rats were administered 0.1 mg of P*17/K4S2(CRM), which contained 0.05 mg of p*17-CRM and 0.05 mg of K4S2-CRM. The control article, CRM, contained 0.065 mg of CRM, which is the average concentration of CRM contained in the vaccine. Intramuscular formulations contained vehicle CAF®01 and i.n. formulations contained vehicle 10 mM Tris.

**TABLE 1:** Toxicology study groups for days 0 and 21 intramuscular administration

Group^*a*^	Test/control article dose (mg/dose)	Animals^*a*^	
		Main	Recovery
CAF®01	0	5M, 5F	5M, 5F
CRM/CAF®01	0.065	5M, 5F	5M, 5F
P*17/K4S2(CRM)/CAF®01	0.1	5M, 5F	5M, 5F

a CRM, cross-reacting material 197; M, male; F, female.

**TABLE 2:** Toxicology study groups for day 42 intranasal administration

Group^*a*^	Test article dose (mg/dose)	Animals^*a*^	
		Main	Recovery
1. Tris	0	5M, 5F	5M, 5F
2. CRM/Tris	0.065	5M, 5F	5M, 5F
3. P*17/K4S2(CRM)/Tris	0.1	5M, 5F	5M, 5F

a The same animals described in Table 1; Tris, 10 mM Tris; CRM, cross-reacting material 197; M, male; F, female.

All study animals were observed throughout treatment until half of the study animals were euthanized and necropsied on study day 46 (main cohort, 4 days after dose 3); the remaining rats were euthanized and necropsied on study day 70 (recovery cohort, 4 weeks after dose 3). There were no unscheduled deaths reported over the course of the study.

There were no vaccine-related findings reported for mortality, clinical observations, body weights, food consumption, ophthalmology, clinical pathology (hematology, coagulation, clinical chemistry, and urinalysis), or gross pathology.

### Local irritation assessment (skin and mucosal Draize scoring).

An evaluation of local skin and mucosal irritation was made at each scheduled dose. All animals were observed pre-dose and then at 3, 24, 48, and 72 h post-dose. Continued observations of up to 7 days post-dose were made if an observation persisted. At the injection site, incidence of erythema and edema were assessed. At the conjunctival or ocular and nasal sites, incidence of erythema, edema, discharge, and blepharospasm (abnormal, involuntary muscular contractions of the eyelids) were assessed.

The first i.m. dose administered on day 0 was to the right thigh (dose site) of all animals. An absence of or minimal erythema at the dose site was observed in all groups. Edema was also assessed, with scores reported in the P*17/K4S2(CRM) group being similar to or lower than the CAF®01 and CRM groups over the assessment period. This observation suggests that the reactions were related to the CAF®01 and/or CRM components and not P*17/K4S2(CRM).

The second i.m. dose administered on day 21 was to the left thigh of all animals. An assessment was made of the left thigh (dose site) and the right thigh (non-dose site). No evidence of local irritation at the non-dose site was observed. As with the first i.m. dose, absence of or minimal erythema and edema at the dose site was observed in all groups. Scores reported in the P*17/K4S2(CRM) group were similar to or lower than the CAF®01 and CRM groups over the observation period. Observations made at the second i.m. dose reinforced what was observed after the first i.m. dose, that local irritation reactions were related to CAF®01 and/or CRM components and not P*17/K4S2.

The third dose administered on day 42 was an i.n. inoculum. Vehicle for this dose schedule was Tris buffer. An assessment was made of the left thigh and the right thighs (non-dose sites). No evidence of local irritation at the non-dose sites was observed. Absence of or minimal conjunctival or ocular erythema and nasal congestion was observed. As with doses 1 and 2, the local reactions in the P*17/K4S2(CRM) group were similar to or lower than the CAF®01 and CRM groups over the assessment period. No other evidence of irritation was noted. The overall findings were that local irritation findings were considered to be related to CAF®01 and/or CRM and not related to P*17/K4S2(CRM).

### Immunogenicity and functional assessment of vaccines.

**FIG 5:**
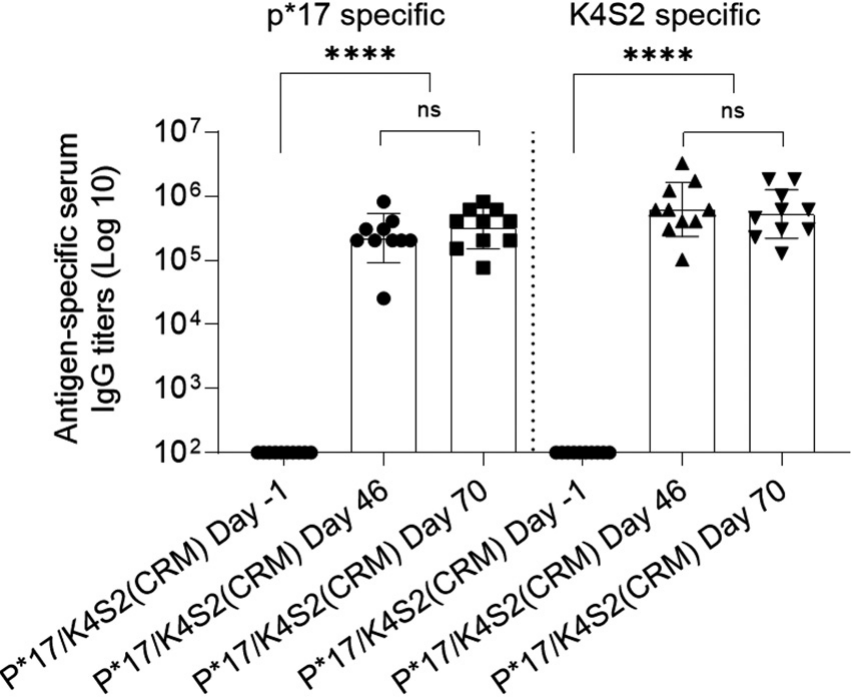
Vaccine peptide-specific serum IgG titers induced in Sprague-Dawley rats after vaccination with P*17/K4S2(CRM). Sprague-Dawley rats were administered an i.m. inoculation of 0.1 mg/dose of P*17/K4S2(CRM)/CAF®01 on days 0 and 21 and an i.n. inoculation of 0.1 mg/dose of P*17/K4S2(CRM)/Tris on day 42. Rats were euthanized at day 46 and day 70. Peptide-specific serum IgG titers (Geomean) are shown for individual rats euthanized at day 46 (*n* = 10; 5 male, 5 female) and 70 (*n* = 10; 5 male, 5 female). Pre-vaccination (day −1) serum IgG titers of the same rats assessed at day 46 or 70 are also shown. The endpoint titer was defined as the highest dilution that gave an absorbance of >3 standard deviations above the mean absorbance of negative control wells. Statistical analysis was performed using a nonparametric, unpaired Mann-Whitney U test (one-tailed) to compare groups (ns, *P* > 0.05; ****, *P* < 0.0001). Analysis was performed and the graph generated in GraphPad Prism 8.1.2.

Immunogenicity of P*17/K4S2(CRM) in the rat model was assessed by ELISA and the endpoint titers reported. Analysis of rat sera collected 4 days after the final dose (day 46) indicated the presence of specific serum IgG antibodies to S. pyogenes antigens p*17 and K4S2. The level of peptide-specific IgG remained similar at day 70 (Fig. 5). Antibodies to p*17 and K4S2 in rat preimmunization serum were not detectable.

### Functional assessment of vaccine-induced antibodies by their ability to recognize S. pyogenes and their capacity to protect interleukin 8 (IL-8) from degradation.

**FIG 6:**
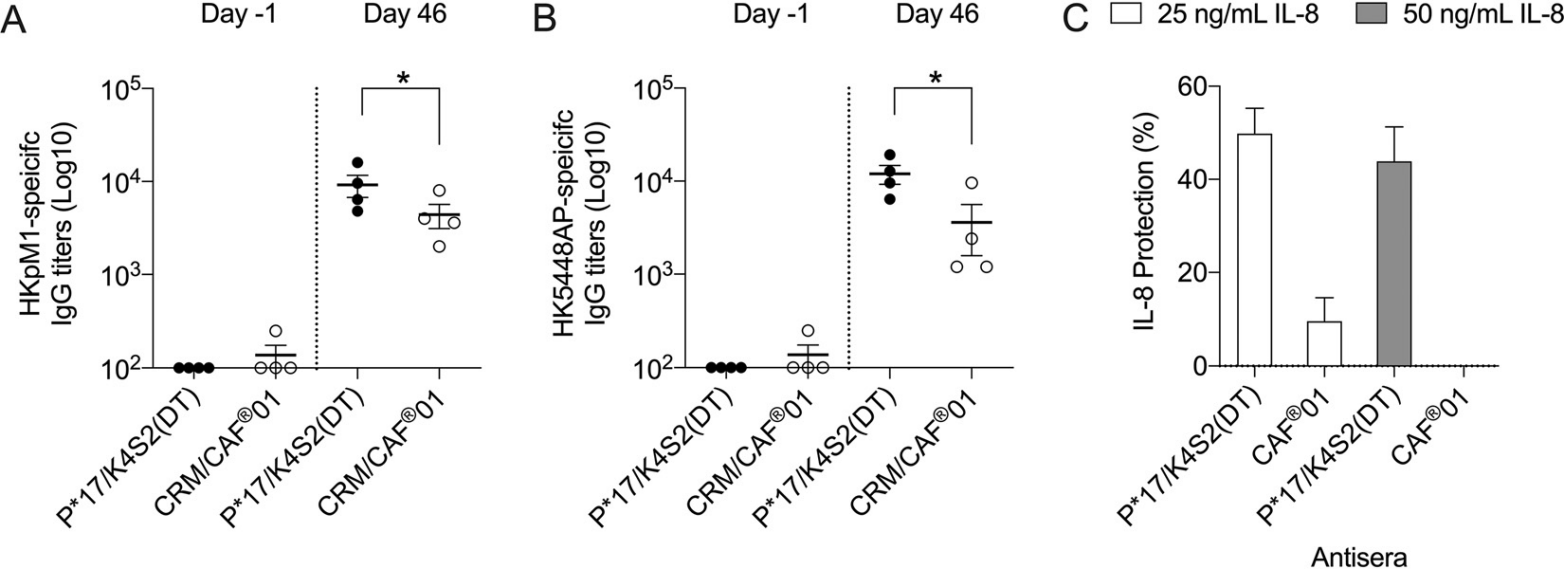
Functionality of vaccine-induced antibodies. (A and B) Binding of P*17/K4S2(CRM) antibodies to S. pyogenes. Nunc MaxiSorp ELISA plates were coated with 200 μg/ml of heat-killed (HK) pM1 and 5448AP. P*17/K4S2(CRM) or CRM/CAF®01 antiserum were assessed. Vaccine antibodies specific for HKpM1 (A) and HK5448AP (B) were detected using HRP-conjugated rat secondary IgG antibody. The endpoint titer was defined as the highest dilution that gave an absorbance of >3 standard deviations above the mean absorbance of negative control wells. Mean ± SEM of individual titers of sera collected from days −1 (*n* = 4) and 46 (*n* = 4) are shown. Statistical analysis was performed using a nonparametric, unpaired Mann-Whitney U test (one-tailed) to compare groups (*, *P* < 0.05). (C) IL-8 protection by vaccine antisera. Cell-free culture supernatant from *covR/S* MT S. pyogenes strain 5448AP was coincubated with 50 ng/ml or 25 ng/ml of rIL-8 and antisera from P*17/K4S2(CRM) or CAF®01 vaccinated mice for 16 h at 37°C. In a parallel experiment, cell-free culture supernatant was preincubated with test antisera prior to coincubation with IL-8. Pooled data from both experiments are shown. Spy-CEP antiserum and naive serum were used as positive and negative controls, respectively. Uncleaved IL-8 was measured by a Quantikine ELISA and is shown as IL-8 protection. The percentage protection values have been normalized to the positive- and negative-control sera (Spy-CEP = 100% and naive = 0%).

Direct antibody recognition of bacterial surface antigens in whole-cell preparations was assessed by ELISA. Sera from the rats collected predosing (day −1) and at main necropsy (day 46) from P*17/K4S2(CRM) and the CRM control (*n* = 4/cohort) were added to wells coated with heat-killed (HK) whole-cell preparations of S. pyogenes *emm* 1 strains pM1 (WT) and 5448AP (MT). Direct binding to bacterial surface proteins was defined by specific IgG titers. Overall, significant binding to bacterial surface proteins was observed in the day 46 titers for both cohorts compared to the day −1 titers. A comparison of binding between P*17/K4S2(CRM) vaccine-induced antibodies and the CRM-induced antibodies was also made. Binding to WT pM1 and the MT 5448AP by P*17/K4S2(CRM) vaccine-induced antibodies was significantly higher (*P* < 0.05) than the CRM-induced antibodies. Antibodies to DT and to CRM have previously been shown to bind to S. pyogenes (20, 21). There was no significant difference in binding between day −1 titers for both cohorts (Fig. 6A and B).

Interleukin-8 (IL-8) protection was assessed in the presence of culture supernatant from S. pyogenes MT strain 5448AP, known to express high levels of the IL-8-degrading protease Spy-CEP. Sera collected at day 46 from P*17/K4S2(CRM) and CRM control cohorts (*n* = 4/cohort) were coincubated with 5448AP and two different concentrations (25 and 50 ng/ml) of recombinant IL-8 (rIL-8). IL-8 percentage protection was defined in comparison to positive (Spy-CEP antisera) and negative (naive) controls. In a parallel experiment, the effect of pre-incubation of culture supernatant with test antiserum, i.e., prior to its coincubation with IL-8, was also assessed. Both experimental protocols resulted in comparable data on IL-8 protection (Fig. 6C): P*17/K4S2(CRM) provided 44 to 50% IL-8 protection with 50 and 25 ng/ml of IL-8, respectively, in comparison to CAF®01 antiserum, which provided less than 10% protection. (Fig. 6C).

### Organ weights.

No vaccine-related weight changes were observed in the adrenal glands, brain, epididymis, kidneys, liver, lungs, spleen, thymus, thyroid gland, or reproductive organs at the day 46 or 70 necropsies. A slight decrease in the heart weights of male and female rats receiving P*17/K4S2(CRM) compared to the vehicle control was observed at the day-46 sacrifice. Although the minimal decrease was statistically significant in males and nonsignificant in females, the heart weight of all rats was within normal weight range. The decrease in heart weight was not observed at recovery necropsy on day 70 and there was no correlating data suggesting toxicity. Therefore, the overall conclusion was that the weight change was incidental and unrelated to P*17/K4S2(CRM) administration.

### Histopathological analysis.

Microscopic observations were reported at similar incidence and severity across all groups and were therefore not considered to be specifically related to administration of vaccine. Observations noted at day 46 were minimal mixed cell infiltration in the nasal cavity, minimal to mild mixed cell infiltration in the larynx, and minimal to mild lymphohistiocytic inflammation at the injection site.

The mixed cell infiltration in the nasal cavity and larynx (lymphocytes, plasma cells, and occasional neutrophils) was noted more in the CRM and vaccine groups than the CAF®01/Tris vehicle group, suggesting that this was a response to intranasal administration of CRM. The cell infiltration was no longer observed at day 70, indicating a complete recovery. At the i.m. injection site, minimal to mild inflammation was noted at a similar incidence and severity across all groups, suggesting that the finding was most likely related to the i.m. injection procedure and possibly the vehicle, CAF®01. This finding was observed at day 70, indicating no recovery. Overall, all findings were considered nonadverse.

### Examination of the brain, heart, joints and kidneys.

The brain and heart of all rats were within normal limits, with no evidence of histopathology observed. There was also no evidence of inflammation in the joints of any animals administered P*17/K4S2(CRM). A spectrum of histopathological findings in the kidneys were reported at both day 46 and 70 necropsies and are outlined in Table 3. The observations were considered to be incidental and not vaccine related. No other evidence of changes to the kidneys was reported.

**TABLE 3:** Histopathology observations in kidney

Pathology	Males (5 per group examined)			Females (5 per group examined)		
	Vehicle	CRM^*b*^	P*17/K4S2	Vehicle	CRM^*b*^	P*17/K4S2
Day 46 observations^*a*^						
Within normal limits (no.)	2	2	3	4	4	4
Cast; minimal (no.)	1	0	0	0	0	0
Chronic progressive nephropathy, minimal (no.)	3	2	2	1	1	0
Chronic progressive nephropathy, mild (no.)	0	1	0	0	0	0
Mineralization, minimal (no.)	0	0	0	0	0	1
Mineralization, mild (no.)	0	0	0	1	0	0
Day 70 observations^*a*^						
Within normal limits (no.)	3	4	3	4	4	4
Chronic progressive nephropathy, minimal (no.)	1	1	1	1	0	1
Chronic progressive nephropathy, mild (no.)	1	0	0	0	0	0
Dilation; tubular, focal, mild (no.)	0	0	2	0	1	0

a Number of animals observed with pathology.

b CRM, cross-reacting material.

## DISCUSSION

We have demonstrated for the first time the efficacy of a S. pyogenes vaccine, formulated with a modern adjuvant that can provide protection against multiple strains, including highly virulent strains, and at both mucosal and skin portals of entry. Administration of a GLP-grade product to male and female rats did not result in any adverse outcomes. S. pyogenes infections and their sequelae lead to significant morbidity and mortality worldwide. Serious S. pyogenes disease fulfils all the World Health Organization (WHO) criteria for listing as a Neglected Tropical Disease (NTD, a heterogeneous group of diseases that affect some of the poorest people on the planet) (22). The WHO has recognized this global burden and issued a call for a S. pyogenes vaccine in a 2018 resolution on RHD. The two common portals of entry of S. pyogenes into humans are skin and the nasopharynx, and infection caused by either can lead to RF/RHD and other severe diseases (23–25). Hence, an effective vaccine would need to prevent infections at both primary sites of S. pyogenes infection.

An early clinical trial in 1978 identified site-specific vaccine efficacy wherein intranasal vaccination was more efficacious at preventing S. pyogenes colonization of the oropharynx than parenteral vaccination (26). Likewise, parenteral immunization with heat-killed S. pyogenes adjuvanted to CAF®01 did not mount a Th17 response in mice lungs and failed to induce protective mucosal immunity (12). We have also demonstrated that systemic immunization with the p*17-related vaccine, J8-DT/Alum, is insufficient for protection against URT S. pyogenes infection (10). However, Penfound and colleagues demonstrated that a vaccine administered subcutaneously and containing amino-terminal epitopes from six common strains could provide protection against mucosal and subcutaneous challenge from two of the organisms whose sequences were represented in the vaccine (27). While this was encouraging, the ability of individuals to respond to multiple epitopes in a polyvalent vaccine may be limited (28). To overcome the constraints of profound strain variation and site-specific immunity, we investigated a prime-pull vaccination strategy, which has been shown to elicit mucosal and systemic immunity (11), with highly conserved vaccine epitopes from two critical virulence factors of S. pyogenes. The two vaccine derivatives (p*17 and K4S2), when conjugated to either DT or its genetically modified analogue CRM, elicited comparable mucosal and systemic immunogenicity and protective efficacy against highly virulent streptococci. These data confirm the comparability of the two carrier molecules, DT and CRM. We have previously shown that epitopes from the C3-repeat region of the M-protein (p*17 and J8) elicit *emm* type independent protective immunity (7, 8, 20).

Carrier-induced epitopic suppression (CIES) is a major concern for the development of peptide-based vaccines that require carrier molecules such as DT to prime the immune response against the immunizing epitope (19). The diphtheria, tetanus, and whooping cough vaccine (DTap) is integrated within the childhood immunization regimen. Therefore, we investigated whether preexisting DT antibodies would affect the immunogenicity of the DT-conjugated CAF®01 vaccine. We showed that priming with DT followed by three doses of P*17/K4S2(DT) resulted in boosting of DT-specific and p*17-sepcific IgG responses. These data support previous findings where priming with DT did not suppress the immunogenicity of a peptide-CRM conjugate vaccine (21). It will also be important to consider the effect of other conjugate vaccines against Haemophilus influenzae type b, Streptococcus pneumoniae, and Neisseria meningitidis, all of which also use CRM as a carrier protein (29). These vaccines contain similar amounts of CRM as the P*17/K4S2(CRM) vaccine described here. Similarly, it will be important to test that vaccination with P*17/K4S2(CRM) will not compromise the response to widely used licensed CRM conjugate vaccines.

We utilized a whole-cell ELISA method to assess the binding of vaccine-induced antibodies to the surface of S. pyogenes. P*17/K4S2(DT) antisera from mice and P*17/K4S2(CRM) antisera from rats both demonstrated strong binding to S. pyogenes. The binding to both *covR/S* WT and MT S. pyogenes isolates were comparable. These findings support our previous data with MJ8CombiVax (J8-DT+K4S2-DT/Alum), where the combination vaccine antisera showed high binding affinity to S. pyogenes (21). As previously reported with DT, we saw binding of CRM-specific antibodies (induced in rats) to S. pyogenes which could be attributed to sequence homology between DT/CRM and S. pyogenes (20). It was also demonstrated that as a result of this homology, DT protein induced cross-reactive antibodies to the M-protein of S. pyogenes and led to low level (∼20%) opsonization of S. pyogenes *in vitro* and low level protection against S. pyogenes challenge *in vivo* (20). Since CRM is closely related to DT (containing a single amino acid substitution in diphtheria toxin), we believe binding of CRM antibodies to S. pyogenes will exert a similar effect and thus enhance the targeted immune response to specific antigen. We also show that P*17/K4S2(CRM) antiserum was able to significantly reduce Spy-CEP-mediated degradation of IL-8. This would enhance neutrophil ingress to the site of infection, leading to opsonophagocytic killing of S. pyogenes (9). We also demonstrated that vaccination with P*17/K4S2(DT) provides enduring immunity. Despite the fact that antigen-specific serum and salivary IgG titers had significantly dropped following 10 weeks of rest, there was >85 and 94% reduction in the URT and systemic bacterial load.

Vaccine studies with CAF®01 in mice (11, 12, 30), minipigs (31), and humans (16) have shown that systemic priming followed by airway boosting promotes the induction of mucosal and serum humoral responses. CAF®01 is a strong Th1/Th17-promoting adjuvant (11). These cell subsets play a critical role in the protection against S. pyogenes (32–36). CAF®01 has also been shown to create a depot effect, leading to increased monocyte influx to the site of injection and the draining lymph nodes (37). On the other hand, aluminum hydroxide (Alum) induces a Th2-type immune response that may suppress the immune mechanisms required for Th1-type cell development (38). The adjuvanting activity of CAF®01 and Alum was assessed in a phase I safety and immunogenicity study of a chlamydia vaccine (16). The CAF®01- and Alum-adjuvanted vaccines were administered 3× i.m. followed by 2× i.n. administrations of unadjuvanted vaccine. The CAF®01 vaccine promoted higher antibody and cell-mediated immune responses than the Alum vaccine, thus selecting the CAF®01-adjuvanted vaccine for further clinical development (16). Mucosal administration of vaccines can lead to systemic unresponsiveness, a phenomenon referred to as oral or mucosal tolerance (39, 40). Avoiding adjuvant delivery to the nasal cavity should avoid immune tolerance and also increase the safety profile of the vaccine.

Supported by these encouraging data from the literature and our own preclinical data, we assessed the safety, toxicity, and immunogenicity profile of the CAF®01-adjuvanted S. pyogenes vaccine candidate P*17/K4S2(CRM) in male and female Sprague-Dawley rats. Following 2 × i.m. and 1 × i.n. dose, there were no vaccine-related effects on mortality, clinical observations, local irritation, body weights, food consumptions, ophthalmology, clinical pathology parameters, and/or anatomic pathology parameters. Non-adverse effects associated with the administration of the vehicle and/or CRM were characterized by edema at the i.m. injection sites and microscopic observations of lymphohistiocytic inflammation at the intramuscular dose sites and mixed infiltration in the nasal cavity and larynx. There was a robust immune response to peptides p*17 and K4S2 in the P*17/K4S2(CRM) main group (day 46 sera) that persisted in the recovery group (day 70 sera). The vaccine-induced antibodies were shown to be functional in *in vitro* assays. In addition to its immunogenicity in BALB/c mice and Sprague-Dawley rats, P*17/K4S2(CRM) was also shown to be immunogenic in HLA DR3-DQ2 humanized mice. This observation further demonstrates that the immune response to P*17/K4S2(CRM) is not restricted by genes of the major histocompatibility complex.

This study marks a significant advance toward the development of a S. pyogenes vaccine that can induce both mucosal and systemic protective immunity. We show that S. pyogenes vaccine antigens p*17 and K4S2, adjuvanted with a human-compatible delivery system, CAF®01, induced protective long-lasting systemic and local humoral immune responses. Outcomes from the toxicology study establish that P*17/K4S2(CRM) is not toxicologically adverse and safe to be delivered to humans in a clinical trial.

## MATERIALS AND METHODS

### Ethics statement and animals.

All animal protocols were reviewed and approved by the Griffith University Animal Ethics Committee (AEC) in accordance with the National Health and Medical Research Council (NHMRC) of Australia guidelines. BALB/c (female, 6 to 8 weeks in age) mice were sourced from Animal Resource Centre, WA. HLA DR3-DQ2 humanized mice (41) were bred in-house at the Griffith University Animal Facility and routinely genotyped for the appropriate transgene(s).

### Murine immunization.

CAF®01 was sourced under a material transfer agreement (MTA) from Statens Serum Institut (SSI), Copenhagen, Denmark. Peptides p*17 and K4S2 were sourced from China Peptides and conjugated to CRM197 in-house. For i.m. immunizations, 100 μl of the CAF®01 preparation (DDA/TDB; 2,500/500 μg/ml) was mixed with 12.5 μg of p*17-DT/p*17-CRM and 12.5 μg of K4S2-DT/K4S2-CRM (total 25 μg of vaccine antigen) at a 1:1 volume ratio. For i.n. immunizations, 12.5 μg of p*17-CRM and 12.5 μg of K4S2-CRM were made up to 30 μl using 10 mM Tris buffer. Female BALB/c mice (4 to 6 weeks old) were immunized i.m. on days 0 and 21 with P*17/K4S2(DT)/CAF®01 or P*17/K4S2(CRM)/CAF®01 and i.n. on day 42 with P*17/K4S2(DT)/Tris or P*17/K4S2(CRM)/Tris. Serum and saliva samples were collected on day 50 for antibody determination by ELISA. For the DT preexposure study, mice were immunized on days 0, 21, and 28 with 25 μg of DT adjuvanted with Alum. Serum samples were collected on days 35 and 70 for antibody determination by ELISA.

### Upper respiratory tract and invasive infections.

Vaccinated and control mice were infected via the URT with S. pyogenes, as previously described (10). Briefly, mice were anaesthetized via an intraperitoneal (i.p.) injection of 100 μl ketamine:xylazil: H_2_O (1:1:10). Once immobilized, the *covR/S* MT strain 5448AP at a concentration of 5 × 10^8^ CFU/ml (5 × 10^6^ CFU/10 μl) was intransally inoculated at 5 μl/nare. Mice were monitored daily for signs of illness as per the score sheet approved by Griffith University Animal Ethics Committee. Mice were sacrificed on day 3 post-infection and nasal-associated lymphoid tissue (NALT) was removed for bacterial burden enumeration.

Mice were challenged via the skin with 1 × 10^8^ CFU/ml of 5448AP, as described elsewhere (8). Briefly, mice were anaesthetized with a mixture of ketamine:xylazil, as above. The fur from the nape of the neck of mice was removed using clippers and a shaver, and the skin was wiped clean with an ethanol swab. Mechanical scarification of the skin was performed using a metal file. Following skin abrasion, S. pyogenes was topically applied. Once the inoculum had completely absorbed on the skin, a temporary cover was applied on the wounded site and mice were housed in individual cages. On day 6 post-infection, mice were sacrificed, and blood was collected for bacterial burden (systemic) enumeration.

### ELISA.

Peptide-specific serum and mucosal IgG was measured by enzyme-linked immunosorbent assay (ELISA) as previously described (10, 42). Briefly, a specific peptide was coated onto Nunc MaxiSorp ELISA plates at 5 μg/ml. Samples were assessed using 2-fold serial dilutions of 1:100 of serum or 1:2 of saliva. Peptide-specific antibodies were detected with horseradish peroxidase (HRP)-conjugated goat anti-mouse-IgG antibody for murine studies (1:3,000 for serum and 1:100 for saliva; Bio-Rad Laboratories) or goat anti-rat-IgG for toxicology studies (1:10,000; Bio-Rad Laboratories). For direct binding to bacteria, Nunc MaxiSorp ELISA plates were coated with 200 μg/ml of heat-killed pM1 (*emm* 1) and 5448AP (*emm* 1). Samples were assessed using 2-fold serial dilutions of 1:100 of serum. Whole cell-specific antibodies were detected using goat anti-mouse IgG antibody (1:3,000) or goat anti-rat IgG antibody (1:5,000). For all assays, antibody detection was by SIGMA*FAST* OPD substrate (Sigma-Aldrich), which was added according to manufacturer’s instructions. Absorbance was measured at 450 nm on a Tecan Infinite M200 Pro plate reader (Tecan Group Ltd.). End-point titer was defined as the highest dilution that gave an absorbance of >3 standard deviations above the mean absorbance of the negative-control wells.

### Interleukin-8 protection assay.

The assay was conducted as previously described, with minor modifications (8). Briefly, S. pyogenes strain 5448AP MT was grown to stationary phase. Cell-free culture supernatant was coincubated with recombinant IL-8 (at 25 or 50 ng/ml concentration) and test antiserum from rats vaccinated with P*17/K4S2(CRM) or CAF®01 for 16 h at 37^°^C. In some experiments, cell-free culture supernatant was preincubated with test antisera prior to coincubation with IL-8. Spy-CEP (GenScript) antiserum and naive serum were used as a positive and negative control, respectively. Uncleaved IL-8 was measured using a Quantikine ELISA kit (R & D Systems). Data were normalized to recombinant Spy-CEP (100%) and naive (0%) sera.

### Toxicology study vaccine and control articles.

Peptides conjugates p*17-CRM and K4S2-CRM were manufactured at clinical grade by Auspep Clinical Peptides (Melbourne, Australia). The lyophilized products were combined and vialled for further processing with CAF®01. Lyophilized GMP grade CRM197 was commercially sourced from Pfenex, USA. The adjuvant CAF®01 was sourced from SSI as a sterile liquid suspension and 250 μl of CAF®01 (DDA/TDB; 2,500/500 μg/ml) was used for rat i.m. immunizations. Sterile 10 mM Tris was supplied by Citoxlab, USA. Prior to dosing test and control articles were prepared as follows: a prescribed volume of CAF®01 or Tris was extracted with a sterile syringe and directly added to vialled lyophilized peptide conjugates or CRM197, vortexed at high speed every 10 min for up to 30 min, and then administered to rats.

### Toxicology study.

The toxicology study was conducted by Citoxlab, USA in compliance with the Food and Drug Administration (FDA) Good Laboratory Practice (GLP) Regulations as set out in Title 21 of the United States Code of Federal Regulations, Part 58. The study was designed with reference to the ICH M3(R2): Guidance on Non-Clinical Safety Studies for the Conduct of Human Clinical Trials and Marketing Authorization for Pharmaceuticals and the FDA Redbook 2000: General Guidelines for Designing and Conducting Toxicity Studies. With the exception of the manufacturing, characterization of the test article, control article, and CAF®01, and the immunogenicity analysis, all procedures were performed by Citoxlab, USA.

### Animals.

Male and female Sprague-Dawley rats were supplied by Charles River Laboratories, Inc. At the onset of dosing, the animals were 9.5 weeks old. The body weights ranged from 189.7 to 250.0 g and from 163.5 to 230.9 g for males and females, respectively. On arrival, all animals were subjected to a health assessment. Animals were group housed in an animal room where the environment was set to maintain a temperature of 20 to 26 °C, a relative humidity of 50% ± 20%, a light/dark cycle of 12 h light/12 h dark (except during study protocol-designated procedures), and a minimum of 10 air changes per hour. All rats were given free and continuous access to food (Purina Mills Certified Rodent Diet 5002 in “meal” form; *ad libitum*) with the exception of fasting prior to scheduled clinical pathology collections and necropsy. All animals were given free and continuous access to tap water. An acclimation period of 15 days was allowed between receipt of the animals and the start of dosing to accustom the animals to the laboratory environment.

### Administration of vaccine doses.

Twenty rats (10 male, 10 female) per group were administered inoculations on days 0, 21, and 42. On days 0 and 21 rats, were administered a 0.25-ml i.m. injection of 0.1 mg P*17/K4S2(CRM)/CAF®01, 0.065 CRM/CAF®01, or CAF®01. On day 42, rats were administered a 0.1-ml i.n. inoculum of 0.1 mg P*17/K4S2(CRM)/Tris, 0.065 CRM/Tris, or 10 mM Tris. For i.m. inoculums, the injection site was shaved free of hair a day prior to each administration. The dose formulation was allowed to come to room temperature, and the 0.25-ml dose volume was administered on day 1 to the right quadricep (thigh muscle) and on day 22 to the left quadricep. For the i.n. inoculum, the dose formulation was allowed to come to room temperature and the 0.1-ml dose volume (0.05 ml/nare) was administered in 0.025 ml increments, alternating nostrils between each increment.

### Assessment of repeat dose toxicity.

Experimental endpoints were moribundity/mortality (at least once daily), clinical signs/physical examinations, inoculation site scoring for reactogenicity, body weights, food consumption, ophthalmology, clinical pathology (clinical chemistry, hematology, coagulation, and urinalysis), organ weights (adrenal glands, brain, epididymis, heart, kidneys, liver, lungs, spleen, thymus, thyroid gland, and reproductive organs), immunogenicity, gross necropsy observations, and histopathology analyses. Microscopic examinations were performed on the aorta, heart, brain, gross lesions, injection sites (left and right quadriceps), kidney, liver, lungs, nasal cavity, olfactory bulb, larynx, pharynx, and trigeminal pathway. All animals were bled prior to dosing and five females and five males of each group were bled prior to each necropsy. Blood samples were collected with EDTA for hematological tests, or without anticoagulant for biochemical tests. Neutral buffered 10% formalin was used for fixation and preservation of tissues. Immunogenicity was performed at Griffith University and all other analyses were performed at Citoxlab, USA.

### Clinical pathology (clinical chemistry, coagulations, urinalysis, and hematology).

Clinical chemistry parameters were sample appearance, creatinine, A/G ratio, globulin, alanine aminotransferase, glucose, albumin, phosphorus, alkaline phosphatase, potassium, aspartate aminotransferase, protein, bilirubin, sodium, calcium, triglycerides, chloride, urea, cholesterol, and gamma-glutamyltransferase. Coagulation parameters were activated partial thromboplastin time and prothrombin time. Urinalysis parameters were bilirubin, blood, color and appearance, glucose, ketones, leukocytes, pH, protein, specific gravity, urobilinogen, and nitrites. Hematology parameters measured were cell morphology, hematocrit, hemoglobin concentration, mean corpuscular volume, mean corpuscular hemoglobin, mean corpuscular hemoglobin concentration, platelet count, red blood cell count, red cell distribution width, reticulocyte counts, white blood cell count, and white blood cell differential.

### Local irritation assessment.

**Dermal assessment.** An evaluation of dermal reactions for erythema and edema (scale 0 to 4) at the i.m. dosing site was performed prior to dosing, and at approximately 3, 24, 48, and 72 h post-dose. In addition, the previous dosing site was assessed at the time of subsequent dosing, and at 24 h post-dose. Scoring continued daily for up to 7 days post-dose administration if erythema and/or edema persisted beyond the last scheduled assessment. Dermal observations were recorded using a modified Draize scoring scheme.

**Ocular and nasal assessment.** An evaluation of ocular and nasal reactions (scale 0 to 2) was performed prior to dosing, and at approximately 3, 24, 48, and 72 h post-dose. In addition, both i.m. injection sites were assessed prior to i.n. dosing and at 24 h post-dose. Assessment parameters were conjunctival edema (none, slight, well defined); conjunctival or ocular erythema (none, slight, well-defined); ocular discharge (none, serous discharge, mucoid discharge); blepharospasm (none, ≤50% closed, >50% closed); nasal discharge (none, serous discharge, mucoid discharge); and nasal congestion (none, slight, well defined). For all evaluations, the scoring continued once daily up to 7-days post-dose administration if a parameter persisted beyond the last scheduled assessment.

### Statistical analysis.

Mean and standard deviations were calculated, as appropriate, for all quantitative data. As applicable, continuous group mean data that were examined statistically were evaluated for equality or homogeneity of variance using the Decision Tree statistical structure. The Decision Tree statistical structure includes analysis of variance (ANOVA), nonparametric analysis of variance, pairwise tests by the Dunnett’s Test for parametric and nonparametric data, simple *t* tests, and the Bartlett’s test for homogeneity of variance. The data were analyzed for dose-related trends using the Williams Test (parametric data) or the Shirley Test (nonparametric data). Nonhomogeneous data were analyzed using a stepwise Dunnett’s Test (parametric data) or a modified Steel Test (nonparametric data). Frequency data (i.e., incidence data, etc.) that were examined statistically were evaluated using the Chi-Square and/or Fisher’s exact tests. Statistical tests were performed as two-sided tests with results taken as significant with probability (*P*) levels of <0.05 or <0.01. Statistical analysis was performed with SAS software. Antibody titer data are presented as geometric mean with geometric standard deviation (SD), with data analysis performed using GraphPad Prism version 8.1.2.

10.1128/mBio.03537-20.1 FIG S1 Humanized HLA mice immunogenicity following vaccination with P*17/K4S2(DT). Download FIG S1, PDF file, 0.01 MB. Copyright © 2021 Ozberk et al. 2021 Ozberk et al. https://creativecommons.org/licenses/by/4.0/ This content is distributed under the terms of the Creative Commons Attribution 4.0 International license.

10.1128/mBio.03537-20.2 TABLE S1 Organs and tissues for histopathological examination. Download Table S1, PDF file, 0.09 MB. Copyright © 2021 Ozberk et al. 2021 Ozberk et al. https://creativecommons.org/licenses/by/4.0/ This content is distributed under the terms of the Creative Commons Attribution 4.0 International license.
