# Supplementary material for: Prime-Pull Immunization with a Bivalent M-Protein and Spy-CEP Peptide Vaccine Adjuvanted with CAF®01 Liposomes Induces Both Mucosal and Peripheral Protection from covR/S Mutant Streptococcus pyogenes
Source: mBio. 2021 Feb 23;12(1):e03537-20. doi: 10.1128/mBio.03537-20 (PMC8545125; doi:10.1128/mBio.03537-20)
Supplement: FIG S1 [file mbio.03537-20-sf001.pdf]

Supplementary Figure

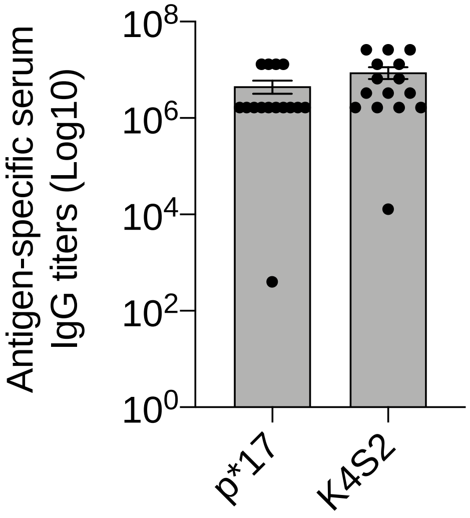

Supplementary Fig. 1. Humanized HLA mice immunogenicity following vaccination with P*17/K4S2(DT). HLA DR3-DQ2-humanized mice (n=15) were immunised via the prime-pull method with P*17/K4S2(DT) (D0-IM with antigen/CAF®01, D21-IM with antigen/CAF®01 and D42-IN with antigen/Tris). Antigen-specific serum IgG levels were assessed in vaccinated mice n=15. The end-point titer was defined as the highest dilution that gave an absorbance of >3 standard deviations above the mean absorbance of negative control wells.
