# Supplementary material for: Prime-Pull Immunization with a Bivalent M-Protein and Spy-CEP Peptide Vaccine Adjuvanted with CAF®01 Liposomes Induces Both Mucosal and Peripheral Protection from covR/S Mutant Streptococcus pyogenes
Source: mBio. 2021 Feb 23;12(1):e03537-20. doi: 10.1128/mBio.03537-20 (PMC8545125; doi:10.1128/mBio.03537-20)
Supplement: TABLE S1 [file mbio.03537-20-st001.pdf]

Supplementary Table

Table S1. Organs and tissues for histopathological examination

Tissue	Organ Weighed	Collected	Microscopic Examination
Adrenal gland	X	X	
Aorta		X	X
Animal Identification		X	
Bone, femur		X	
Bone with marrow (sternum)		X	
Brain [f]	X	X	X
Cecum		X	
Cervix		X	
Colon		X	
Duodenum		X	
Epididymis	X	X	
Esophagus		X	
Eye		X	
Gross lesions		X	X
Harderian gland		X	
Heart	X	X	X
Ileum		X	
Injection Sites (Right and Left Quadriceps)		X	X
Jejunum		X	
Kidney	X	X	X
Larynx		X	X
Liver	X	X	X
Lung with large bronchi	X	X	X
Lymph node (mandibular)		X	
Lymph node (mesenteric)		X	
Mammary gland		X	
Muscle (gastrocnemius)		X	
Nasal Cavity (4 levels)		X	X
Nerve, sciatic		X	
Olfactory Bulb		X	X
Optic nerve		X	
Ovary	X	X	
Oviduct		X	
Pancreas		X	
Parathyroid gland		X	
Peyer's Patch		X	
Pharynx		X	X
Pituitary gland	X	X	
Prostate	X	X	

Table S1. Organs and tissues for histopathological examination

Tissue	Organ Weighed	Collected	Microscopic Examination
Salivary gland		X	
Seminal vesicle		X	
Skin/subcutis		X	
Spinal cord		X	
Spleen	X	X	
Stomach		X	
Testis	X	X	
Thymus	X	X	
Thyroid gland	X	X	
Tongue		X	
Trachea		X	
Trigeminal Pathway		X	X
Urinary bladder		X	
Uterus	X	X	
Vagina		X	

X: activity carried out
